# The limits of awareness campaigns: persistent age-disparate relationships in KwaZulu-Natal more than a decade after the 2012 sugar daddies destroy lives campaign

**DOI:** 10.3389/ijph.2026.1610222

**Published:** 2026-09-03

**Authors:** Sbonelo Charles Chamane, Lehlogonolo Makola, Sean Jooste, Sinothando Dlamini, Sizulu Moyo, Nompumelelo Zungu

**Affiliations:** 1 Public Health, Societies and Belonging, Human Sciences Research Council, Pretoria, South Africa; 2 School of Public Health, University of the Witwatersrand Johannesburg, Johannesburg, South Africa; 3 Family Medicine Department, School of Medicine, University of Pretoria, Pretoria, South Africa; 4 University of Cape Town School of Public Health, Observatory, South Africa; 5 School of Nursing and Public Health, University of KwaZulu-Natal, Durban, South Africa

**Keywords:** adolescent girls and young women, age-disparate relationships, public health campaigns, South Africa, structural determinants

## Highlights

- Age-disparate relationships remain common among adolescent girls and young women in KwaZulu-Natal more than a decade after the Sugar Daddies Destroy Lives campaign.
- Awareness campaigns alone are insufficient to reduce age-disparate relationships when structural drivers such as poverty, unemployment, and gender inequality remain unaddressed.
- Long-term, multi-sectoral interventions combining economic empowerment, education, adolescent-friendly services, and social norm change are needed to reduce HIV risk and teenage pregnancy.

## Introduction

In 2012, the KwaZulu-Natal (KZN) Department of Health launched a comprehensive public health campaign “Sugar Daddies Destroy Lives” discouraging adolescent girls and young women from forming relationships with substantially older men due to teenage pregnancy and the persistently rising HIV rates [1]. A study conducted in KZN reported that women reporting an age-disparate partnership in any of their three most recent relationships were 58% more likely to test HIV-positive [2]. These relationships often reflect power imbalances, economic dependence and are associated with teenage pregnancy and reduced ability to negotiate safer sexual practices [3, 4]. KZN, which continues to shoulder a disproportionate share of South Africa’s HIV epidemic, has long targeted age-disparate partnerships in prevention efforts [1, 5]. The 2012 “Sugar Daddies Destroy Lives” campaign had hundreds of billboards and an accompanying public-information push that sought to reframe relationships with older men as shameful and dangerous rather than desirable, linking sexual choices to HIV risk and social harm. The campaign intended to shift social norms, increase risk awareness, and protect young women from exploitative partnerships. It was a classic public-health messaging intervention: visible, inexpensive relative to structural programmes, and designed to change behaviour through information and stigma.

## Current trends in age-disparate relationships

**FIGURE 1:**
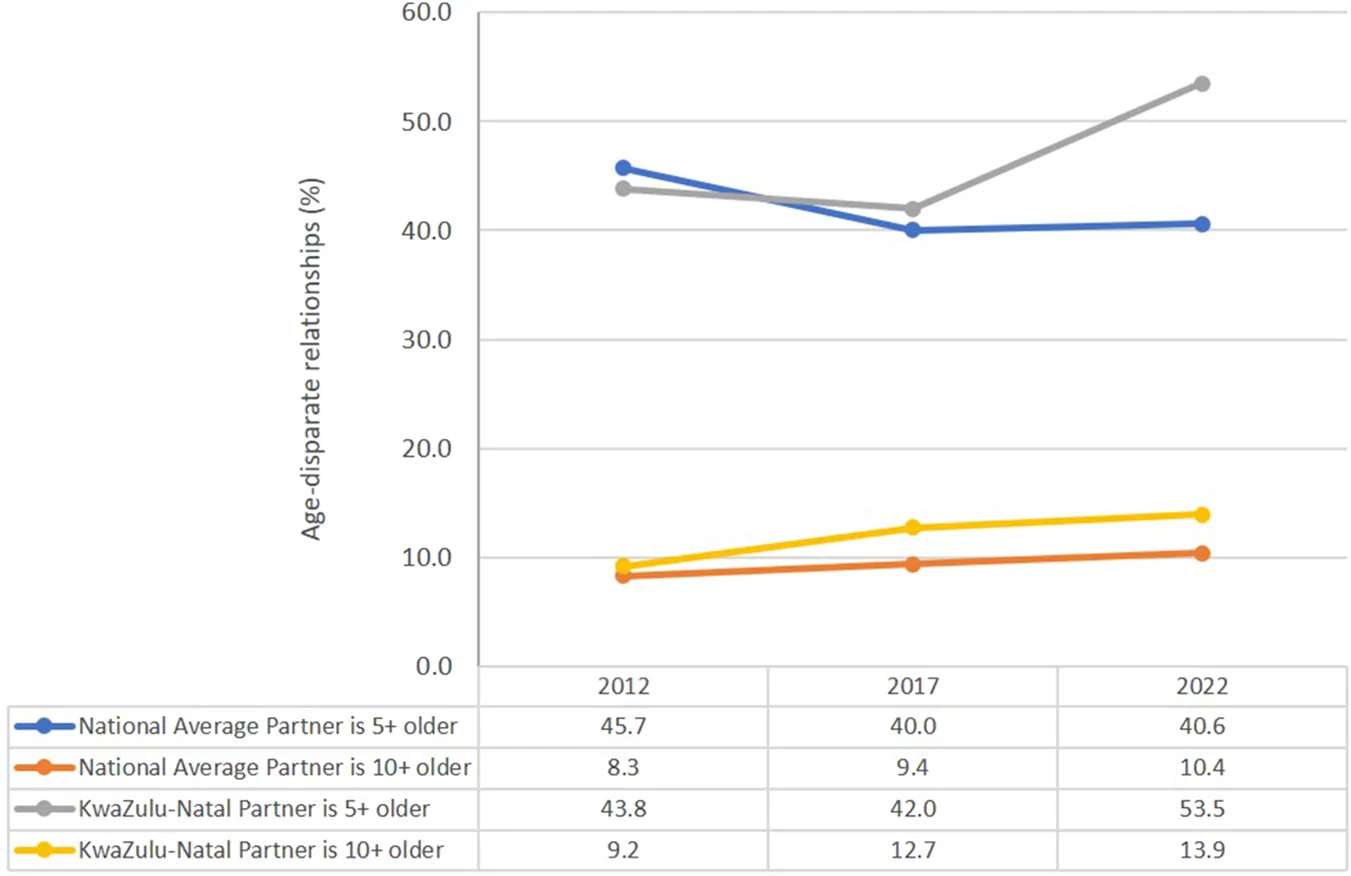
Trends in age-disparate relationships among females aged 15–24 Years in South Africa and KwaZulu-Natal, 2012–2022 [5].

What do trends in the data show? Population based survey data from three South African National HIV Prevalence, Incidence and Behaviour Surveys reveal a worrying pattern among females aged 15–24 in KwaZulu-Natal [5]. The proportion reporting partners at least 5 years older was 43.8% in 2012, 42.0% in 2017, and rose to 53.6% in 2022 (Figure 1). For relationships with partners at least 10 years older, the percentage rose from 9.2% in 2012, to 12.7% in 2017, and 13.9% in 2022. Far from a sustained decline after the campaign, KZN recorded a rise in five-year age gaps by 2022, and consistently exceeded the national average across survey rounds. In practical terms, by 2022, more than one in two young women in the province reported a partner five or more years older. This prevalence should alarm policymakers and programme designers alike.

## Understanding the limits of awareness campaigns

If the problem persists, why did the 2012 campaign not produce a lasting change? The answer is that awareness interventions confront behaviour, but not always the structural conditions that drive it. For many young women in KZN, relationships with older men are embedded in broader structural realities such as entrenched poverty, long-standing youth unemployment rates, limited access to education and secure livelihoods, and social norms that enable and valorise male provision. Transactional exchanges for school fees, transport, housing, or basic needs are often pragmatic responses to economic precarity rather than expressions of ignorance about HIV. Messaging can alter perceptions, but cannot, on its own, substitute for the material alternatives that make safer choices feasible. Literature has pointed to the importance of addressing the broader structural drivers of age-disparate and transactional relationships, including poverty, gender inequality, and economic vulnerability [6].

## Evidence for multi-sectoral prevention strategies

In Zimbabwe, targeted peer-support approaches for pregnant adolescents and adolescent mothers have shown positive effects when combined with comprehensive sexuality education and adolescent-friendly health services. The Champions of Change peer-based approach used trained adolescent mother mentors to deliver tailored sessions, improving sexual and reproductive health rights knowledge, attitudes, healthcare-seeking behaviours, and trust in health services [7]. In Kenya, data has shown that cash-transfer programmes and interventions promoting school attendance can contribute to reductions in early sexual initiation and teenage pregnancy, although adolescent pregnancies remain closely linked to age-disparate sexual relationships, with the majority of men who fathered children with teenage girls reported to be aged 20–29 years (89.7%) [8, 9]. Similarly, findings from South Africa suggest that cash transfers may strengthen condom use, reduce risky partnerships, and improve HIV testing by increasing young women’s economic confidence and decision-making power [10].

Together, the findings from Ethiopia further demonstrate that teenage pregnancy resulting from age-disparate relationships is shaped by broader social, educational, economic, and health-service factors. Sifer et al. highlighted limited reproductive-health discussions alongside social, cultural, educational, and economic influences, while Nyamhanga and Luoga emphasised the protective role of education and the importance of access to adolescent-friendly sexual and reproductive health services [11, 12]. These findings reinforce the need for interventions that extend beyond awareness messaging to address the structural and service-related factors shaping adolescent sexual and reproductive health outcomes.

The common lesson is that multi-sectoral interventions combining education, economic opportunity, and social norm change address both motivations and constraints that shape young women’s relationships. These, however, should be well embedded into societal structures to increase access and uptake and must be sustained long term. This is not an argument against public messaging. Campaigns like “Sugar Daddies Destroy Lives” can raise awareness, spark debate, and help de-stigmatise seeking social support services. But the KZN experience illustrates the limits of billboard-driven prevention when structural drivers remain fully unaddressed.

### Conclusion

More than a decade after the launch of the Sugar Daddies Destroy Lives campaign, age-disparate relationships among adolescent girls and young women in KwaZulu-Natal remain widespread, with the proportion reporting partners at least 5 years older increasing from 43.8% in 2012 to 53.6% in 2022. Rather than demonstrating a sustained reduction in risk, these trends suggest that awareness campaigns alone are insufficient to change behaviours that are deeply rooted in poverty, gender inequality, youth unemployment, and economic dependence. This does not diminish the value of public health messaging. Awareness campaigns can increase knowledge, stimulate public dialogue, and support shifts in social norms. However, their impact is likely to be limited unless they are accompanied by sustained investments in interventions that address the structural conditions shaping young women’s choices. Evidence from South Africa and elsewhere indicates that economic empowerment, school retention initiatives, cash-transfer programmes, comprehensive sexuality education, adolescent-friendly health services, and peer-led support can complement awareness campaigns by expanding opportunities and strengthening young women’s agency. Such approaches are essential if South Africa is to achieve meaningful reductions in teenage pregnancy, new HIV infections among adolescent girls and young women, and the broader inequalities that continue to fuel vulnerability.
